# Welcoming Novelty in the Treatment of Coronary Artery Disease: FDA Approves the Use of Drug‐Coated Balloon for Angioplasty

**DOI:** 10.1002/clc.70515

**Published:** 2026-11-02

**Authors:** Abdul Haseeb Hasan, Muhammad Ali Abid, Abubakar Nazir

**Affiliations:** ^1^ Department of Medicine King Edward Medical University Lahore Pakistan; ^2^ Department of Medicine The Jewish Hospital‐Mercy Health Ohio USA

**Keywords:** CAD, coronary artery disease, DCB, drug‐coated balloon, NSTEMI, Paclitaxel, STEMI

## Abstract

**Background:**

Coronary artery disease (CAD) remains a leading cause of morbidity and mortality in the United States, with in‐stent restenosis (ISR) posing a significant challenge following percutaneous coronary intervention (PCI). Drug‐eluting stents (DES) have been widely used to mitigate ISR; however, until recently, no drug‐coated balloon (DCB) catheter had been approved for use in the United States. The US Food and Drug Administration (FDA) has now approved AGENT DCB, a paclitaxel‐coated balloon catheter, offering a novel approach to treating ISR.

**Hypothesis:**

The approval of AGENT DCB represents a pivotal advancement in PCI, providing an effective alternative to traditional balloon angioplasty for ISR.

**Methods:**

This article is an editorial review and does not involve original research, statistical analysis, or software‐based evaluations. The findings and discussion are based on the results of the AGENT IDE trial and related literature on the efficacy of DCB catheters in the treatment of ISR.

**Results:**

The AGENT IDE trial, a randomized, multicenter study involving 600 participants across 40 US sites, demonstrated that the paclitaxel‐coated balloon group had a significantly lower target lesion failure rate at 12 months compared to the uncoated balloon group (17.9% vs. 28.6%, *p* = 0.003). The reduction was primarily driven by a lower incidence of target lesion revascularization (13.0% vs. 24.7%, *p* = 0.001) and myocardial infarction related to the target vessel (5.8% vs. 11.1%, *p* = 0.02). Additionally, no cases of stent thrombosis were observed in the DCB group compared to 3.2% in the control group (*p* = 0.001), reinforcing the device's safety and efficacy.

**Conclusions:**

The FDA approval of AGENT DCB marks a significant milestone in the management of ISR, offering a promising alternative to conventional interventions. With its ability to deliver antiproliferative therapy while avoiding the complications associated with permanent stents, DCB may revolutionize PCI strategies in the United States. Further studies assessing the long‐term impact of this technology, as well as its application in broader patient populations, will be essential to solidify its role in clinical practice.

## Graphical abstract

The US FDA has approved the use of drug‐coated balloons (DCBs) for treating in‐stent restenosis, a major challenge in coronary artery disease management. The AGENT IDE Trial, a multicenter randomized study, evaluated the efficacy of the paclitaxel‐coated balloon catheter, demonstrating reduced rates of target lesion failure, myocardial infarction, and revascularization compared to uncoated balloon angioplasty. This milestone introduces an effective alternative to mitigate restenosis risks following stent placement.

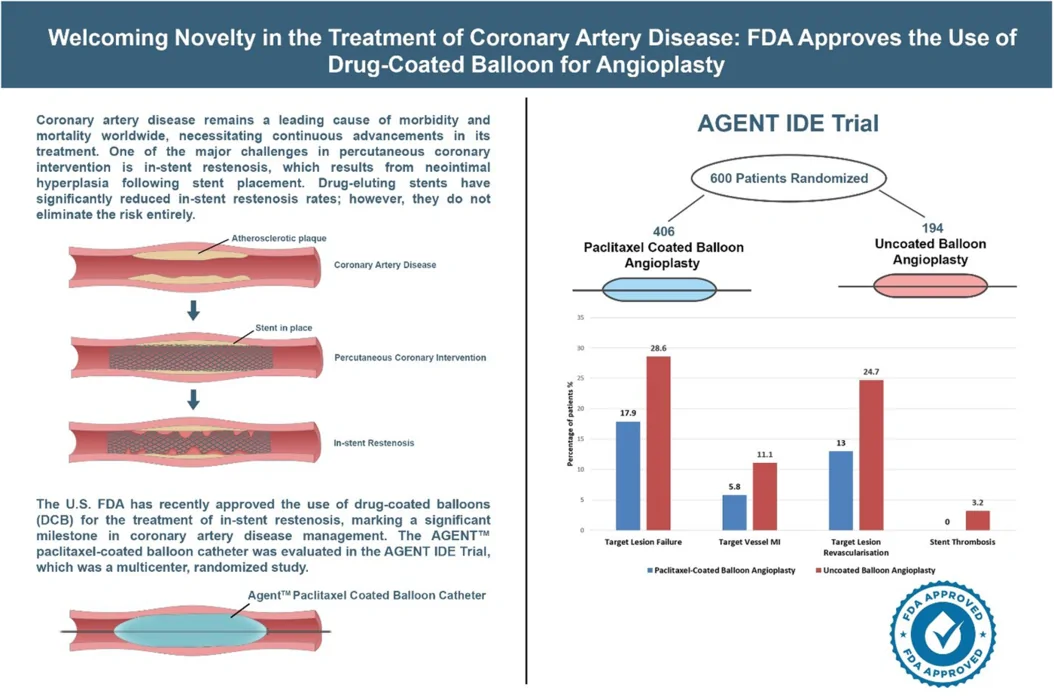

Coronary artery disease (CAD) is a disease entity in which there is a gradual narrowing of the blood vessels supplying blood to the heart (coronary blood vessels) due to the deposition of plaque, which is a buildup of fatty substances [1]. This narrowing leads to a decrease in blood supply to the myocardium, which is the contractile part of the heart and relies highly on the oxygen provided by the blood for its operation [1, 2]. Furthermore, plaques can throw off thrombi that can become lodged in the coronary arteries, which can result in the development of acute coronary syndrome (ACS) [1]. ACS can present as unstable angina, non‐ST‐elevation myocardial infarction (NSTEMI), or ST‐elevation myocardial infarction (STEMI) [1]. One in 20 individuals over the age of 20 suffers from CAD, which is currently the most common type of heart disease in the United States [3, 4].

One of the non‐surgical methods for treating CAD is percutaneous coronary intervention (PCI), formerly known as coronary angioplasty with stent [5, 6]. This process involves guiding the balloon catheter to the site of coronary artery occlusion under radiographic control [5]. The balloon catheter is inflated, and it pushes the atherosclerotic plaque against the inner wall of the vessel [5, 6]. This opens up the obstruction of the blood flow through the vessel and improves blood supply to the heart [6]. The placement of a stent in the vessel is necessary to overcome the vessel recoil as the balloon is deflated, but the presence of the stent itself, which has to stay there permanently, becomes the cause of inflammation [6, 7]. The inflammation promotes fibrin deposition, myofibroblast migration and proliferation, leading to in‐stent restenosis (ISR), which requires repeated interventions to keep the artery patent at the rate of about 1%−2% per year [7, 8]. To overcome this challenge, drug‐eluting stents (DES) were invented. The DES technology is composed of drug coatings with antiproliferative properties, which help prevent patients from developing ISR [6, 8, 9, 10]. Although this is a breakthrough technology, no drug‐coated balloon (DCB) catheter had been approved for use in the United States before [11].

The US Food and Drug Administration (FDA) recently approved DCB, which aims to provide a safe and effective treatment option for ISR and reduce its recurrence [12]. Following this announcement, the AGENT DCB produced by Boston Scientific Corporation was granted approval by the FDA for the treatment of patients with coronary ISR [12]. AGENT DCB consists of a paclitaxel‐coated balloon catheter that delivers a therapeutic dose of the drug (2 μg/mm^2^) to the wall of the vessel, which prevents the neointimal proliferation of the vascular wall after stent placement [13]. The decision was made after the successful completion of the AGENT Investigational Device Exemption (IDE) trial, which yielded positive results in favor of AGENT DCB [11, 14].

AGENT IDE trial is a randomized, multicenter trial which took place at 40 sites across the United States [11]. The trial included 600 participants who were randomly assigned in a 2:1 ratio to undergo angioplasty with either a paclitaxel‐coated balloon (*n* = 406) or an uncoated balloon (*n* = 194) [11]. The primary outcome of the trial was the target lesion failure at the 12‐month mark [11]. The definition of target lesion failure included cardiac mortality, the requirement for a target lesion revascularisation (TLR) procedure, or myocardial infarction relative to the target vessel [11]. The incidence of target lesion failure for the paclitaxel‐coated balloon group against the uncoated balloon group was 17.9% and 28.6% (*p* = 0.003), respectively [11]. The value was highly significant, and it was driven by a ∼50% reduction in both total TLR (13.0% vs. 24.7%; *p* = 0.001) and the myocardial infarction of the target vessel (5.8% vs. 11.1%; *p* = 0.02) [11]. Moreover, stent thrombosis was not seen in any patient in the DCB group when compared to the uncoated balloon group (0% vs. 3.2%; *p* = 0.001) [11]. These results highly support the superiority of the AGENT DCB catheter over traditional balloon angioplasty in patients within the American population.

DCB has been in use in international markets such as Europe, Asia Pacific, and Latin America for a while, but its use has only now been approved in the United States. The use of DCB in the treatment of CAD is expected to yield better results and reduce the load of percutaneous interventions carried out to address ISR. This will decrease the incidence of myocardial infarction occurring due to ISR and improve the long‐term prognosis for patients with CAD. Although patients have shown reluctance to participate in clinical trials in the post‐COVID‐19 era [15], the rapid registration of patients in this trial study highlighted the clinical need of practitioners across the US for this device, who had never used this tool before. Further studies exploring specific population subgroups would be a valuable addition to the existing knowledge supporting AGENT DCB and could deepen our understanding of its role in treating ISR. The approval of DCB for ISR may pave the way for further applications of DCB, such as long diffuse vascular disease and native coronary arteries without stents. The true potential of DCB in coronary circulation still needs to be explored and incorporated into daily practice.

In conclusion, staying aligned with medical advancements and integrating them into routine practice is essential for enhancing patient outcomes. However, such innovations should only be adopted after strong evidence confirms their efficacy and safety, except when the potential benefits of trial implementation significantly outweigh the risks of exclusion. The approval of AGENT DCB demonstrates the FDA's openness to embrace innovation when it is supported by strong scientific evidence. This sets a precedent for future advancements to emerge with compelling data, paving the way for their inclusion in patient treatment regimens.

## Data Availability

Data sharing is not applicable to this article as no data sets were generated or analyzed during the current study.
